# Conventional histological and cytological staining with simultaneous immunohistochemistry enabled by invisible chromogens

**DOI:** 10.1038/s41374-021-00714-2

**Published:** 2021-12-28

**Authors:** Larry E. Morrison, Mark R. Lefever, Heather N. Lewis, Monesh J. Kapadia, Daniel R. Bauer

**Affiliations:** Roche Diagnostics Solutions (Ventana Medical Systems, Inc.), Tucson, AZ 85755 USA

**Keywords:** Assay systems, Diagnostic markers, Prognostic markers

## Abstract

Conventional histological stains, such as hematoxylin plus eosin (H&E), and immunohistochemistry (IHC) are mainstays of histology that provide complementary diagnostic information. H&E and IHC currently require separate slides, because the stains would otherwise obscure one another. This consumes small specimen, limiting the total amount of testing. Additionally, performing H&E and IHC on different slides does not permit comparison of staining at the single cell level, since the same cells are not present on each slide, and alignment of tissue features can be problematic due to changes in tissue landscape with sectioning. We have solved these problems by performing conventional staining and IHC on the same slide using invisible IHC chromogens, such that the chromogens are not visible when viewing the conventional stain and the conventional stain is excluded from images of the IHC. Covalently deposited chromogens provided a convenient route to invisible chromogen design and are stable to reagents used in conventional staining. A dual-camera brightfield microscope system was developed that permits simultaneous viewing of both visible conventional stains and invisible IHC chromogens. Simultaneous staining was demonstrated on several formalin-fixed paraffin-embedded tissue specimens using single and duplex IHC, with chromogens that absorb ultraviolet and near infrared light, followed by H&E staining. The concept was extended to other conventional stains, including mucicarmine special stain and Papanicoulou stain, and further extended to cytology specimens. In addition to interactive video review, images were recorded using multispectral imaging and image processing to provide flexible production of color composite images and enable quantitative analysis.

## Abstract (web summary)

Conventional histological and cytological stains, including hematoxylin & eosin and Papanicolaou, are combined with immunohistochemistry and simultaneously evaluated on the same specimen slide by use of invisible chromogens. Visible and invisible light used to illuminate the specimen are separated and directed to color and monochrome cameras, respectively, providing conventional stain and immunohistochemistry in side-by-side videos on the computer monitor. Alternatively, multispectral imaging of single microscope fields provides archiving and further evaluation utilizing image processing.

Hematoxylin plus eosin (H&E) is the most common histological stain and provides one of the most important cancer diagnostics^1,2^. Pathologists gain an incredible amount of information from H&E stained tissues that supports diagnosis, prognosis, and prediction of therapeutic response^3–5^. Other conventional brightfield stains, such as Papanicoulou (PAP) staining of cervical specimens, and special stains such as giemsa, elastic, mucicarmine, and trichrome stains have long histories of valued use^6,7^. Another brightfield staining technique, immunohistochemistry (IHC), provides a means to stain specific molecular species, commonly proteins, through highly specific antibody reagents, and in combination with H&E, have greatly strengthened the pathologist’s diagnostic capability^8^. As an example, H&E can establish the presence of NSCLC, but often expression of two or more of the proteins p40, thyroid transcription factor-1 (TTF-1), cytokeratins 5 & 6, and/or Napsin A are required to clearly classify the cancer as squamous cell carcinoma (SSC) or adenocarcinoma (ADC)^9,10^.

Unfortunately, complete analysis of a particular tumor by H&E and IHC may require more tissue than is available from a biopsy, especially for needle biopsies and fine needle aspirates. This is because one slide is required for H&E to make the primary diagnosis, and one additional slide is required for each IHC stain, by common clinical practice. IHC multiplexing can increase the number of IHC staining reactions per slide, but still requires at least one slide in addition to the H&E stained slide. De-staining the H&E slide followed by IHC is possible, but complete removal of hematoxylin and eosin may prove difficult and time consuming. Furthermore, comparison of the same specimen regions for each requires the cumbersome process of imaging after H&E and again after IHC, and locating and aligning the regions of interest.

Even when sufficient sample for several slide preparations is available, coordinating the evaluation of the H&E and IHC protein expression patterns is problematic. For tissues, the best case is performing the H&E and IHC on adjacent (serial) tumor sections. However, tissue morphology changes with distance through the tumor specimen, and even serial sections can show significant change in the shape and orientation of various tissue and cellular features. Since cells on one section are not present, or are only partially present on the serial section, exact alignment of H&E and IHC information is rarely possible. The situation is worse for cytology specimens, for which alignment of information between two different specimen slides is not possible.

Multiplexing H&E staining with IHC on the same slide is a solution to the above problems of limited specimen and alignment of information retrieved from different specimen slides. Good H&E staining following single and duplex IHC has been demonstrated^11^. However, while H&E staining of cells and regions surrounding the IHC stain is interpretable, H&E stain overlapping the IHC is obscured. Lighter application of the IHC stains would result in the H&E stain obscuring the IHC stains. Figure 1A shows the absorbance spectra of an H&E-stained formalin-fixed paraffin-embedded (FFPE) tonsil tissue section (solid line) and two other common conventional stains. Also plotted is the human light-adapted (photopic) visual response (relative luminosity factors; dotted line)^12^ showing that the H&E stain absorbs across the entire visible range where conventional IHC chromogens, such as 3,3ʹ-Diaminobenzidine (DAB), Fast Red, and Fast Blue, absorb light. Use of immunofluorescence in place of IHC is also problematic since eosin is strongly fluorescent and hematoxylin can quench fluorescence.

**Fig. 1:**
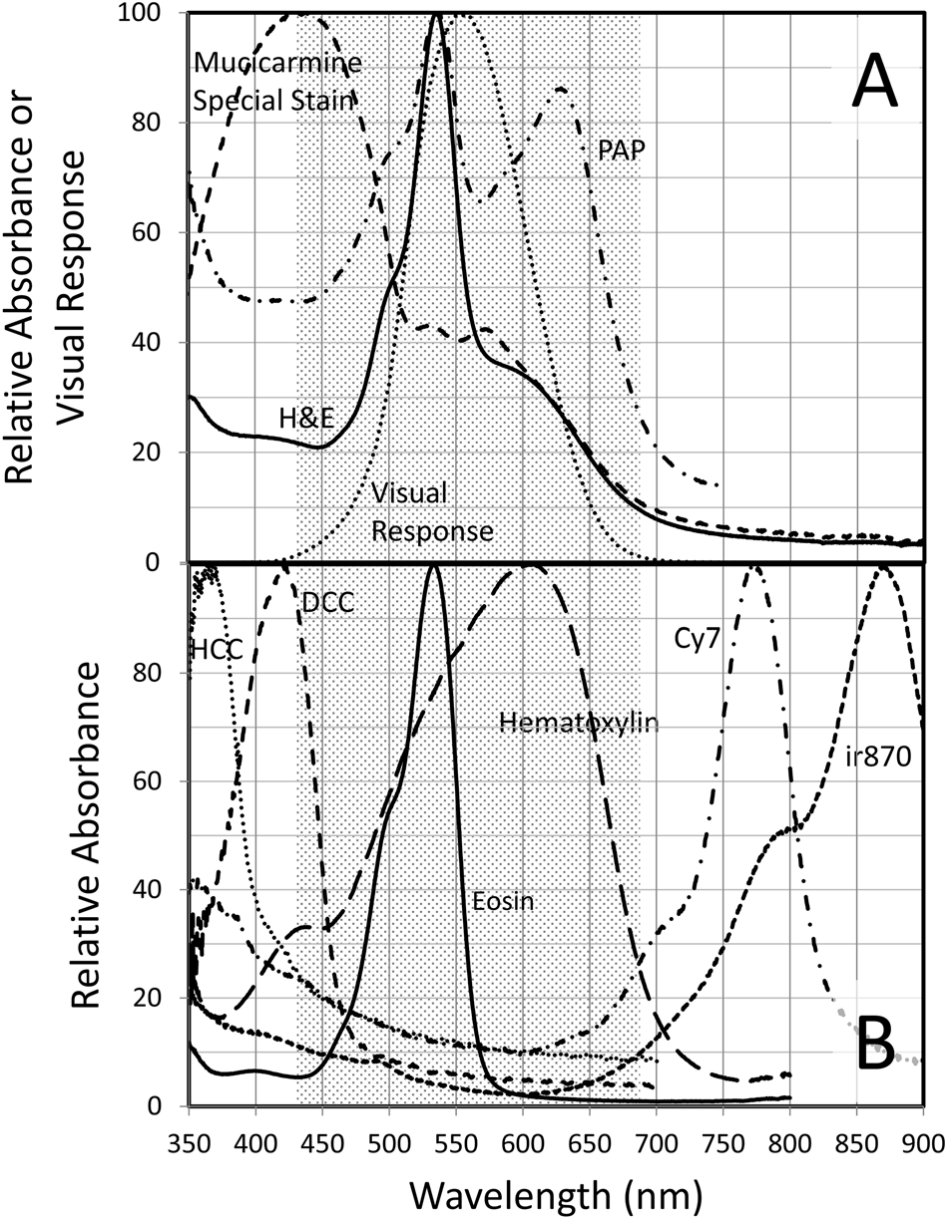
Absorbance spectra of several conventional histological and cytological stains and several invisible chromogens compared to the human photopic (light-adapted) visual response (relative luminosity factors^12^). **A** H&E (tonsil FFPE tissue), PAP (cervical cell ThinPrep), and mucicarmine special stain (NSCLC FFPE tissue) absorbance spectra, and relative luminosity factors, all scaled to 100 at their respective maxima. **B** HCC, DCC, Cy7, and ir870 CDC absorbance spectra (deposited by Ki67 IHC on tonsil FFPE tissue), and hematoxylin and eosin absorbance spectra (separately applied to tonsil FFPE tissue), all scaled to 100 at their respective maxima. The shaded area approximates the visible spectral region, or more specifically, where relative luminosity factors are >1% of maximum.

In this work we demonstrate how the above problems are alleviated by multiplexing ‘invisible’ IHC with H&E simultaneously on the same specimen slide. Invisible chromogens are defined for the present purpose as dyes deposited by enzymatic action, like conventional chromogens, but having absorbance in the ultraviolet (UV) or near infrared (NIR) predominantly outside the boundaries of human visual response. Looking at the absorbance spectra plotted in Fig. 1A, H&E absorbance declines outside the region of visual response, and is significantly reduced below 450 nm and especially above 700 nm. The invisible parts of the light spectrum are unexplored by conventional chromogen development since essentially all brightfield clinical assays are evaluated visually by pathologists. By using invisible chromogens, H&E and IHC stains are spectrally separate, so both types of stain can be present at the same time on the same specimen with minimal interference. We have taken advantage of a new class of chromogens, the covalently deposited chromogen (CDC), which simplifies chromogen development and permits dyes with essentially any desired spectral characteristics to be rapidly converted to chromogens suitable for IHC^13^, including the invisible CDC. We describe a two-camera brightfield microscopy system comprising a color camera and a monochrome camera that permits viewing video of light transmitted by both H&E stain and invisible chromogens, side-by-side or overlaid, in real-time, allowing manual scanning and interpretation in the usual manner of an anatomical pathologist, with one slide taking the place of two or more. In addition to video presentation, the system allows multispectral imaging for archiving and quantitative evaluation, with potential extension to whole slide scanning. We also demonstrate extension to cytology specimens and other conventional stains, including PAP and mucicarmine special stain.

## Materials and methods

### Chromogens

Methods for synthesizing CDCs have been previously described^13–16^. Tyramide-derivatized alkyne click chemistry partner dibenzocyclooctyne (DBCO) and azide-derivatized dye synthesis methods have also been described^17^. Results presented here include azide-derivatives of 7-hydroxycoumrin-3-carboxylate (HCC), 7-diethylaminocoumarin-3-carboxylate (DCC), Cy7, and ir870, a novel derivative of a chloro-Cy7. Synthesis of ir870 is described in the Supplementary Methods document.

### Specimens

FFPE slide-mounted sections from normal (relative to cancer) tonsil, normal (relative to cancer) pancreas, colon cancer, and NSCLC, of anonymized patients were prepared by Ventana Medical Systems, Inc. (VMSI) histology personnel from blocks obtained from the VMSI specimen bank. FFPE slide-mounted sections of mouse xenografts of human breast tumor cell line Calu-3 were obtained from VMSI (Tucson, AZ; Dual ISH 3-in-1 Xenograft Slides, Cat. No. 783–4422). ThinPrep (Hologic, Inc., Marlborough, MA) slides of anonymized High Grade Squamous Intraepithelial Lesion (HSIL) cervical cytology specimen pools were prepared by VMSI personnel from the VMSI specimen bank. All specimens were anonymized and consented to meet all regulatory requirements.

### IHC, Immunocytochemistry (ICC), and conventional staining

Primary antibodies, IHC reagents, and automated staining instruments were obtained from VMSI, including anti-synaptophysin (cat no. 790–4407), anti-CD20 (cat no. 760–2531), anti-CD3 (cat no. 790–4341), anti-CD8 (cat no. 790–4460), anti-HER-2/neu (cat no. 790–2991), anti-Ki-67 (cat no. 790–4286), CINtec *PLUS* Cytology (includes anti-Ki-67 and anti-p16; cat no. 760–100), anti-p40 (cat no. 790–4950), and anti-TTF-1 (cat no. 790–4756). Enzyme-antibody conjugates used with the CDCs were OmniMap anti-mouse HRP (RUO), DISCOVERY (VMSI Cat# 760–4310), and OmniMap anti-rabbit HRP (RUO), DISCOVERY (VMSI Cat# 760–4311). Semi-automated multiplexed detection was performed on a DISCOVERY Ultra system using the listed primary antibodies and detection reagents. The DISCOVERY Universal Procedure was used to create a protocol for the single biomarker IHC and multiplex IHC (details are provided in the Supplementary Methods document). Conventional histological and cytological staining were performed manually following IHC, including H&E, PAP, and mucicarmine special stain (refer to Supplementary Methods document for details). Slides were mounted with Richard Allan Scientific Cytoseal XYL (ThermoFisher Scientifc, Kalamazoo, MI) covering with a type 1.5 coverslip, or mounted on a Sakura Finetek USA (Torrance, CA) Tissue-Tek Film Automated Coverslipper.

### Microscope and imaging systems

**Fig. 2:**
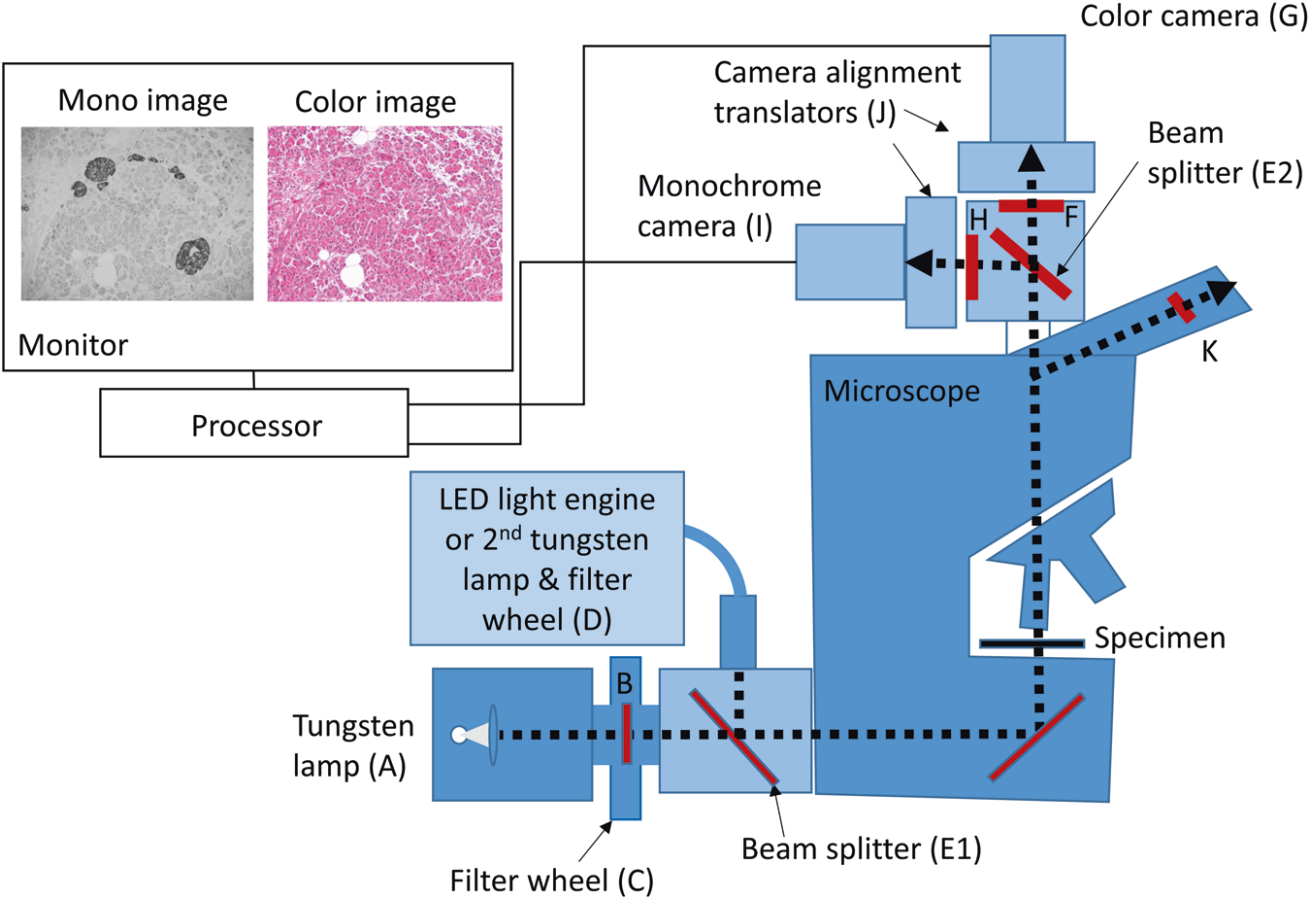
Schematic drawing of dual-camera microscope system. Components include tungsten lamp (**A**) with filters (**B**) mounted in filter wheel (**C**) to pass broadband visible light or narrow visible light bands, invisible light source(s) comprising LED light engines and/or filtered tungsten lamp (**D**), beamsplitter (E1) to combine visible and invisible light, beamsplitter (E2) to separate visible and invisible light and direct to color and monochrome cameras, respectively, filter (**F**) to further exclude invisible light from color camera (**G**), filter (**H**) to further remove visible light from monochrome camera (**I**), camera translators for camera alignment (**J**; part of Thorlabs dual camera mount), and filters to prevent invisible light from reaching eye (**K**).

Referring to the schematic in Fig. 2, a dual-camera brightfield microscope system was designed for simultaneous viewing of both conventional visible stains and invisible IHC chromogens while manually scanning a specimen. Olympus BX-51 and BX-63 microscopes (Olympus, Waltham, MA) were adapted by replacing the conventional microscope lamp and camera. At the microscope lamp port, a dichroic beamsplitter (E1) combined visible illumination, from a tungsten halogen microscope lamp (A), with UV light and NIR light, from light emitting diodes (LEDs) or a second tungsten halogen lamp (D) with optical filtering to provide illumination channels at wavelengths near chromogen absorbance maxima. Light transmitted through the specimen was collected at the objective and split into the visible and invisible components with a second dichroic beamsplitter (E2) within a dual-camera mounting device (Thorlabs, Newton NJ USA) mounted at the camera port. Visible light was directed to a digital color camera (G) and the invisible light was directed to a monochrome camera (I). Additional filtering of light directed to the color and monochrome cameras was provided by filters F and H, respectively. Both cameras used the same underlying 2448 × 2048 pixel CMOS sensor, thereby permitting precise alignment of conventional stain and IHC images. Video from the two cameras was presented side-by-side or overlaid on the computer monitor for simultaneous viewing while examining the specimen (Thorcam software, Thorlabs). Visible light also could be viewed directly through the oculars, with the precaution of mounting bandpass filters (K) within each eyepiece to block invisible light from reaching the eye. Alternatively, the oculars could be replaced with a tube lens that prevents direct viewing. More details of the dual-camera microscope system are provided in the Supplementary Methods document.

Multispectral images for quantitative analysis and composite image formation were recorded on the monochrome camera as previously described^18^. For multispectral imaging of both visible and invisible light channels, the dichroic beamsplitter in the dual-camera mount was replaced with a 100% reflective mirror to direct all light to the monochrome camera, and monochrome camera filters (H) were removed. Image processing was used to create color composite images and perform spectral unmixing as previously described^18^.

### Spectroscopic measurements

Absorbance spectra of deposited chromogens and conventional stains were recorded on slide-mounted specimens placed on the stage of an Olympus BX-63 microscope under illumination with an Olympus 75 W xenon microscope lamp. Transmitted light was measured between 350 and 800 nm in ~0.5 nm increments using a Pryor Scientific Inc. (Rockland, MA) Lumaspec 800 power meter, or measured between 200 and 1100 nm with the Lumaspec power meter upgraded with an Ocean HDX UV to NIR spectrometer (Ocean Optics, Orlando, FL). The spectrum of light transmitted through a stained region of the slide was divided by the spectrum transmitted through an unstained (no tissue) region to provide the transmittance (T) spectrum, which was converted to the chromogen absorbance (A) spectrum using the relationship A = −log_10_T.

## Results

To characterize the CDCs used in this work, absorbance spectra were recorded on tonsil FFPE tissue stained by Ki-67 IHC using the HCC, DCC, Cy7, and ir870 CDCs individually, and plotted in Fig. 1B. Plotted for comparison are the absorbance spectra of tonsil FFPE tissue stained separately with eosin and hematoxylin. The visible range, approximated as 430 nm to 690 nm (relative luminosity factor greater than 1% of the peak value; see Table I in reference 12) is shaded in the figure. While portions of the indicated CDCs lie in the visible region, the majority of their absorbance lies outside the visible, and visual sensitivity is very low over most of the CDC absorbance range.

**Fig. 3:**
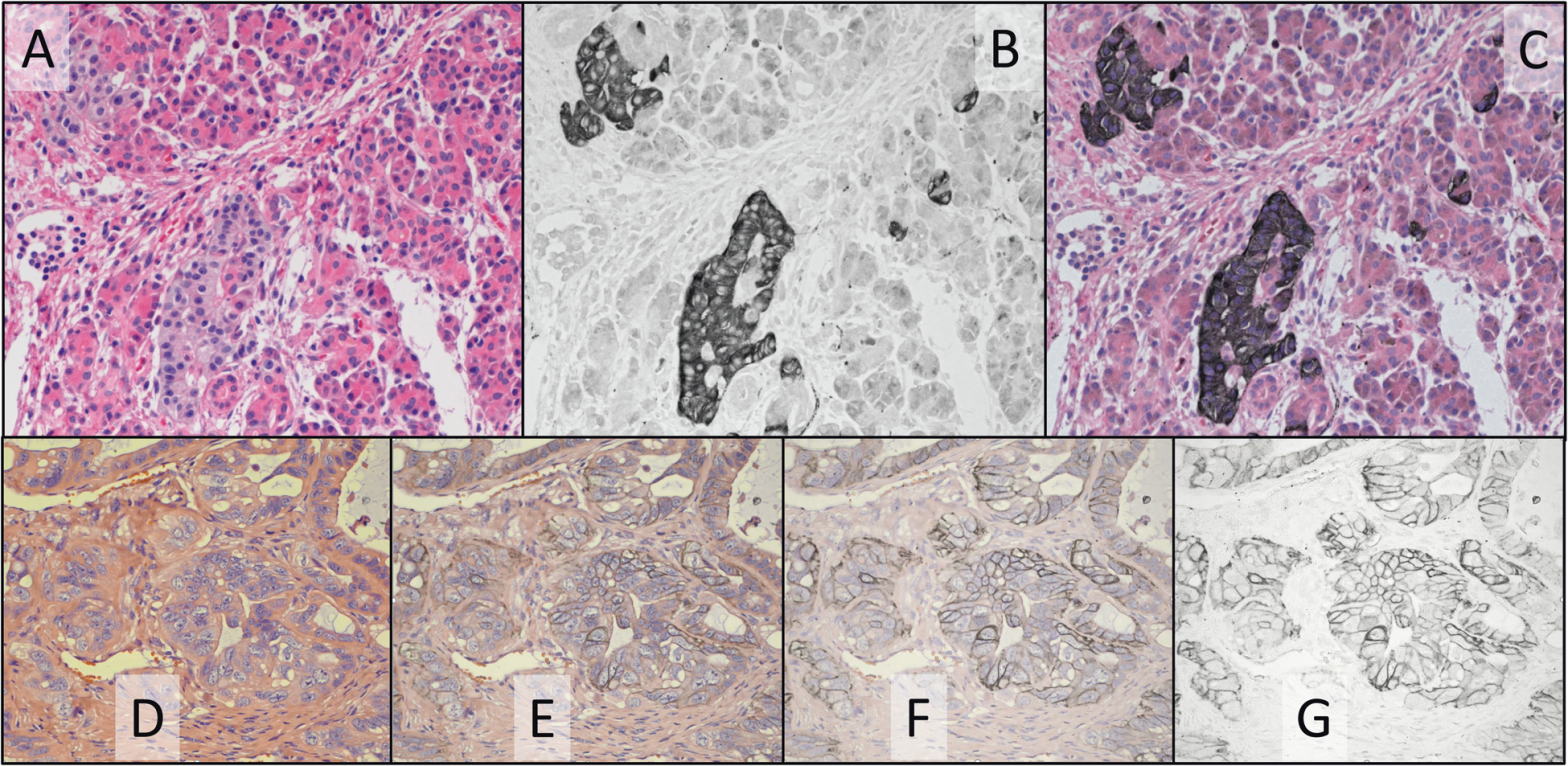
Simultaneous H&E and IHC staining of normal pancreas FFPE tissue for synaptophysin (A–C) and breast tumor xenograft FFPE tissue for HER2 (D–G). **A** Color camera image of transmitted visible light showing H&E staining of pancreas FFPE tissue. **B** Monochrome camera image of transmitted light at 769 nm (filtered tungsten light) where Cy7 CDC absorbs light, staining synaptophysin. **C** Color composite image created as product of color (**A**) and monochrome (**B**) images to show H&E and chromogen (synaptophysin) staining of pancreas tissue simultaneously. Overlays of color (**D**) and monochrome (**G**) images of decreasing color image opacity to show H&E and chromogen (HER2) staining of breast tumor xenograft simultaneously, with selectable relative contribution of color and monochrome images. Images were recorded using a ×20 objective.

To test the ability to combine invisible IHC with H&E staining, IHC targeting the protein synaptophysin was performed on normal pancreatic FFPE tissue followed by conventional H&E staining. The IHC utilized a CDC comprising the NIR absorbing dye Cy7 (absorbance maximum = 774 nm). Using the dual-camera microscope system, the specimen was simultaneously illuminated with broadband visible light from one tungsten lamp and a band of light centered at 769 nm from a second tungsten lamp. Visual examination through the oculars of the microscope revealed normal H&E staining of the specimen, with the absence of synaptophysin staining, while video of both color H&E and invisible chromogen images were presented side-by-side on the computer monitor. Figure 3 shows images of transmitted light recorded on the dual-camera microscope system, with the color camera (Fig. 3A) sensing the visible light and reproducing the view through the oculars, and the monochrome camera (Fig. 3B) sensing the 769 nm light, showing the synaptophysin staining in the absence of H&E stain. Multiplying the two images pixel-by-pixel provides a composite image showing both stains (Fig. 3C). A video overlay of the two images can also be presented by the camera software with selectable weighting of the color H&E and monochrome IHC images. Several levels of weighting are shown in Fig. 3 D–G for FFPE tissue of a human breast tumor cell line (Calu-3) mouse xenograft stained simultaneously with H&E and HER2 IHC using Cy7 CDC. Overlay weighting is varied from 100% color image (Fig. 3D), to 100% monochrome image (Fig. 3G), and two intermediate weightings of the color and monochrome images (Figs. 3E and 3F). In these images, the invisible light was provided with an LED centered at 770 nm. Note that in specifying light channels, ‘769 nm’ will be used in reference to filtered tungsten illumination (filter center wavelength =769 nm) and ‘770 nm’ will refer to LED illumination (nominal center wavelength = 770 nm). Either illumination channel provided similar results and choice of illumination depended upon microscope configuration for a particular experiment.

**Fig. 4:**
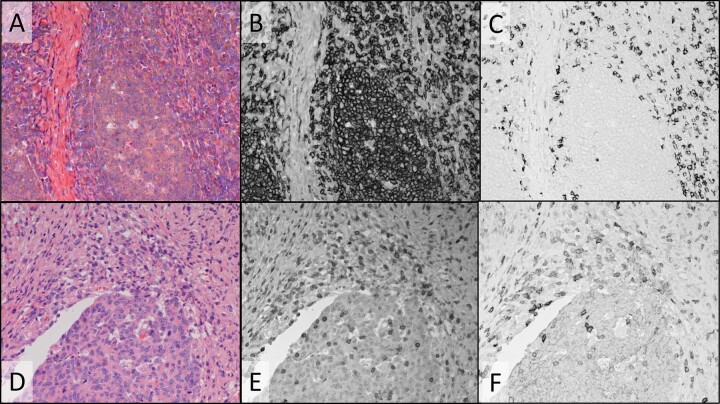
H&E and dual IHC staining of tonsil for CD20 & CD8 (A-C) and colon tumor for CD3 and CD8 (D-F). **A** and **D** Color camera images of transmitted visible light showing H&E staining. **B**, **C** Monochrome camera images of transmitted light at 405 nm (filtered tungsten lamp) where DCC CDC absorbs light, staining CD20 (**B**), and 769 nm (filtered tungsten lamp) where Cy7 CDC absorbs light, staining CD8 (**C**). Monochrome camera images of transmitted light at 385 nm (LED) where HCC CDC absorbs light, staining CD3 (**E**), and 770 nm (LED) where Cy7 CDC absorbs light, staining CD8 (**F**). Images were recorded using a ×20 objective.

Multiplexed invisible IHC with H&E staining was demonstrated by performing sequential IHC targeting CD20 and CD8 on FFPE tonsil tissue followed by H&E staining. CD20 was stained with a DCC CDC (absorbance maximum = 420 nm), and CD8 was stained with the Cy7 CDC. Absorbing in the far blue near the edge of visual perception, DCC can appear weakly yellow by eye with heavy staining, but is barely perceptible with moderate staining and proper filtering in the presence of the H&E stain. Figure 4A–C displays dual-camera images recorded with the color camera, showing the H&E staining (Fig. 4A), and monochrome camera, showing CD20 staining with DCC CDC (Fig. 4B) and CD8 staining with Cy7 CDC (Fig. 4C). The specimen was illuminated with broadband visible light from a tungsten lamp to illuminate the H&E stain while switching between two filters on a second tungsten lamp, centered at 405 nm, to reveal the CD20 stain, and 769 nm, to reveal the CD8 stain.

Multiplex IHC with H&E staining was applied to FFPE colon tumor tissue, targeting the general T-cell marker CD3, and activated T-cell marker CD8. CD3 was stained with the HCC CDC (absorbance maximum = 365 nm) and CD8 was stained with the Cy7 CDC. Figure 4D–F displays dual-camera images recorded with the color camera, showing the H&E staining (Fig. 4D), and monochrome camera, showing CD3 staining with HCC CDC (Fig. 4E) and CD8 staining with Cy7 CDC (Fig. 4F). The specimen was illuminated with broadband visible light from a tungsten lamp to illuminate the H&E stain while switching between two LEDs, centered at 385 nm, to reveal the CD3 stain, and 770 nm, to reveal the CD8 stain.

**Fig. 5:**
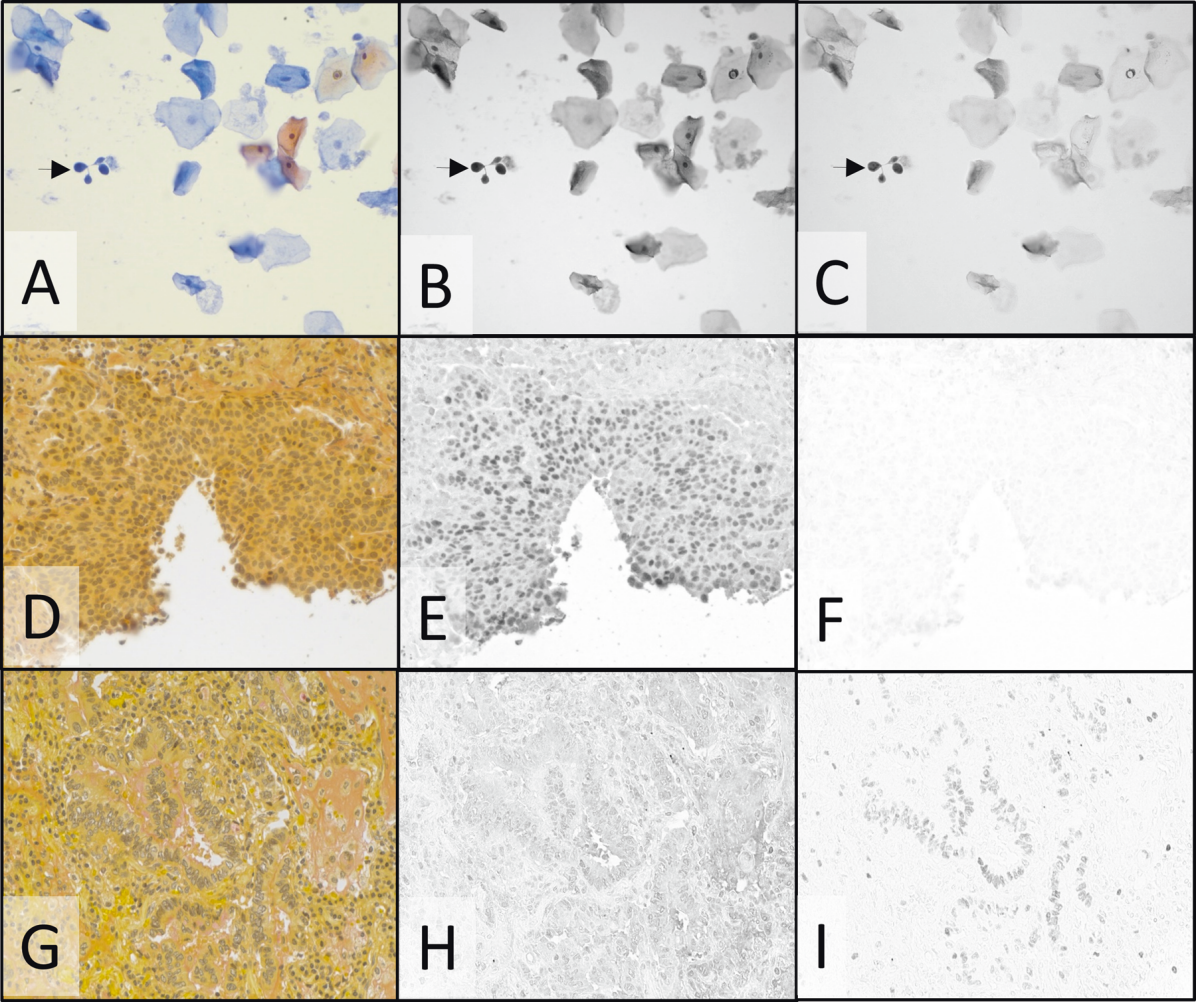
Cytological stain and special stain plus duplex IHC: PAP-staining of cervical cytology specimen (A–C) and mucicarmine special staining of NSCLC SCC (D–F) and ADC (G–I). Color camera images of transmitted visible light showing PAP (**A**) and mucicarmine special stain (**D** & **G**) staining. Monochrome camera images of transmitted light at 405 nm and 770 nm where DCC (**B**) and Cy7 (**C**) absorb light, staining Ki-67 (**B**) and p16 (**C**). Monochrome camera images of transmitted light at 769 nm where Cy7 CDC absorbs light, staining p40 (**E** and **H**), and 880 nm where ir870 CDC absorbs light, staining TTF-1 (**F** and **I**). Images were recorded using a ×20 objective.

Simultaneous staining with IHC was extended to other conventional stains and to cytology specimens. The popular cytology stain, PAP, also showed reduced UV and NIR absorbance (Fig. 1A) that permits staining with invisible chromogens. Cervical cytology specimens were prepared by ThinPrep and stained by IHC with two markers for abnormal cervical cells, Ki-67 using the DCC CDC and p16 using the Cy7 CDC, followed by PAP staining. In conjunction with the broadband visible light, sequential illumination of the two CDCs was provided by 405 and 770 nm LEDs. Figure 5 shows the color PAP image (Fig. 5A), the 405 nm Ki67 image (Fig. 5B), and the 770 nm p16 image (Fig. 5C). An arrow identifies a cluster of cells stained darkly with both IHC markers.

Extension to special stains used mucicarmine special staining of NSCLC tissue and dual IHC for the SCC marker p40 and the ADC marker TTF-1. The Cy7 CDC and ir870 CDC (absorbance maximum = 869 nm) were used for p40 and TTF-1, respectively, to avoid mucicarmine special stain absorbance below 420 nm (Fig. 1A). Figure 5D-I shows images recorded on SCC (D-F) and ADC (G-I) tissue after application of the dual IHC and special stain. Color camera images of the special stain (Fig. 5D, G), 770 nm images of p40 (Fig. 5E, H) and 880 nm images of TTF-1 (Fig. 5F, I) show the proper tissue specificity.

**Fig. 6:**
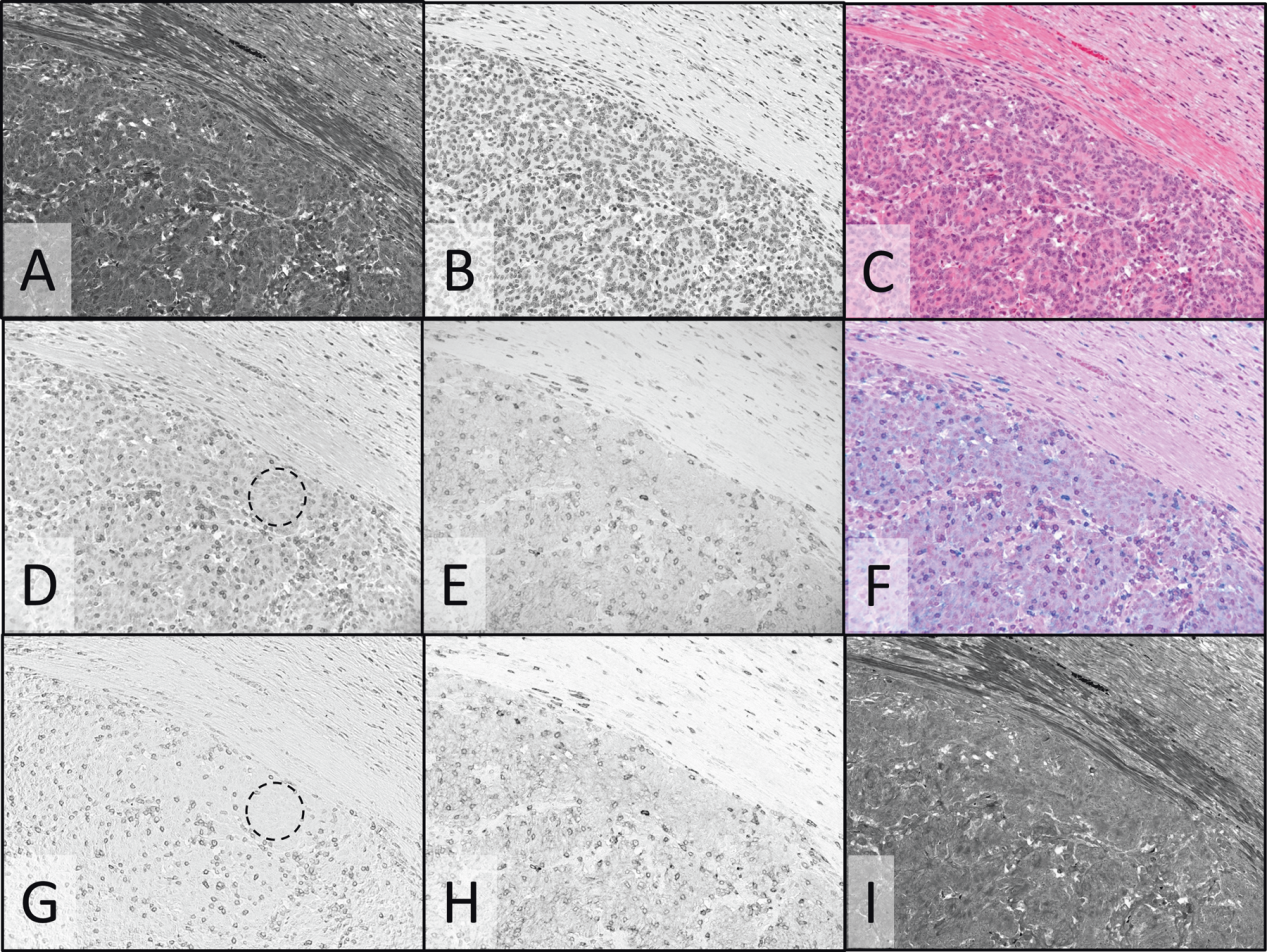
Multispectral imaging and image processing: composite color images and spectral unmixing in colon tumor FFPE tissue stained with CD3/CD8 duplex IHC and H&E. Monochrome images of transmitted light at 513, 620, 390, and 770 nm where eosin (**A**), hematoxylin (**B**), HCC CDC (**D**), and Cy7 (**E**) primarily absorb light, respectively, staining the two components of the H&E stain (**A** & **B**), CD3 (**D**) and CD8 (**E**). Spectrally unmixed images of CD3 (**G**), CD8 (**H**), and eosin (**I**). **C** Color composite image formed from the eosin (**A**) and hematoxylin (**B**) monochrome images. **F** Color composite image formed from the CD3 image (**D**), pseudo-colored magenta, and the CD8 image (**E**), pseudo-colored cyan. Dashed circles in (**D**) and (**G**) emphasize regions with faint hematoxylin crosstalk (**D**) that is removed after unmixing (**G**). Images were recorded using a ×20 objective.

In addition to real-time viewing on the dual-camera microscope system, as used in the above examples, multispectral imaging was used to evaluate single fields of view, using only the monochrome camera and sequential illumination with narrowband invisible and visible light channels provided by LEDs and/or filtered tungsten light. Image processing was applied to produce color composite images of the conventional stains, and to spectrally unmix images to remove spectral crosstalk^18^. Fig. 6 shows images of a different field of the same colon tumor FFPE specimen pictured in Fig. 4D-F (H&E plus CD3/CD8 duplex IHC). The specimen was illuminated sequentially and imaged using four LEDs: 513 nm (predominantly eosin absorbance, Fig. 6A), 620 nm (primarily hematoxylin absorbance, Fig. 6B), 390 nm (primarily HCC CDC absorbance, Fig. 6D), and 770 nm (Cy7 absorbance, Fig. 6E). The color H&E image (Fig. 6C) was created from the 513 nm and 620 nm images and faithfully reproduced the view through the oculars. The color image in Fig. 6F was created by combining the CD3 image, pseudo-colored magenta, and the CD8 image, pseudo-colored cyan. CD8 co-expression with CD3 (activated T-cells) produced blue coloration by subtractive color mixing. Spectral unmixing was applied to the four monochrome images to produce the unmixed CD3, CD8, and eosin images (Fig. 6 panels G-I, respectively) to remove spectral crosstalk. In particular, faint hematoxylin staining seen in the eosin image (Fig. 6A) and CD3 image (Fig. 6D) was eliminated in the unmixed eosin (Fig. 6I) and CD3 (Fig. 6G) images. Comparison of the dashed circles in Fig. 6, panels D and G points out the crosstalk removal. The hematoxylin unmixed image (not shown) and CD8 unmixed image (Fig. 6H) were minimally altered by unmixing since there was minimal crosstalk of the other dyes into the 620 nm hematoxylin and 770 nm Cy7 channels. Since crosstalk is minimal, residual background staining in the Cy7 (CD8) image is likely due to non-specifically bound or adsorbed antibodies, adsorbed Cy7 CDC, or low level CD8 expression.

**Fig. 7:**
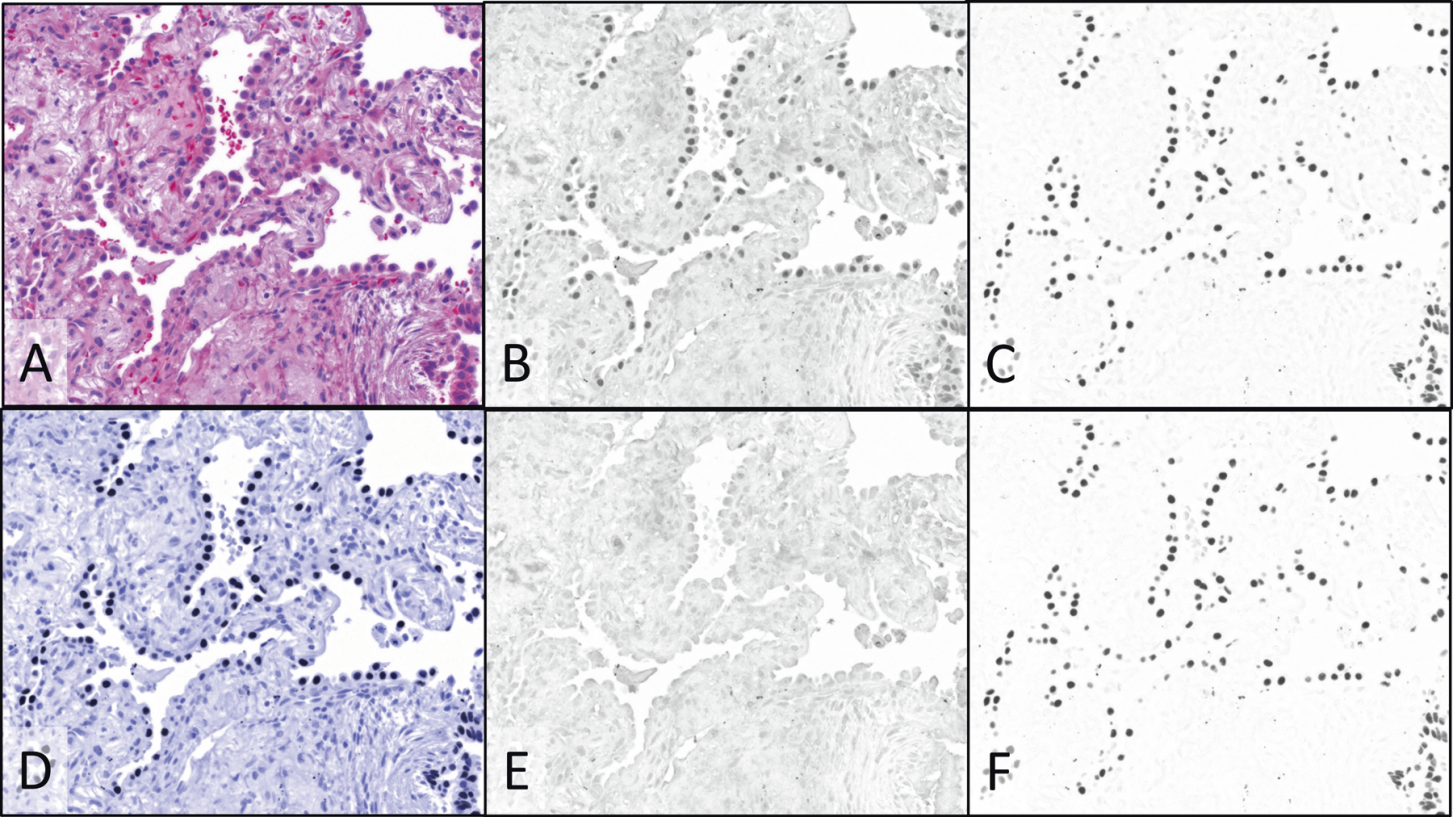
Multispectral imaging and image processing: composite color images and spectral unmixing in NSCLC ADC FFPE tissue stained with p40/TTF-1 duplex IHC and H&E. **A** Color composite image formed from spectrally unmixed monochrome transmitted light images recorded at 510 nm, where eosin primarily absorbs light, and 599 nm, where hematoxylin absorbs light (monochrome images not shown). Monochrome camera images of transmitted light at769 nm where Cy7 CDC primarily absorbs light, staining p40 (**B**), and 880 nm, where ir870 CDC absorbs light, staining TTF-1 (**C**). Spectrally unmixed images of p40 (**E**) and TTF-1 (**F**). **D** Two-color composite image formed from the spectrally unmixed hematoxylin (not shown) and TTF-1 (**F**) images. Images were recorded using a ×20 objective.

Figure 7 shows images of NSCLC ADC FFPE tissue stained with H&E, p40 IHC using the Cy7 CDC, and TTF-1 IHC using the ir870 CDC, illuminated with four filtered tungsten lamp channels: 510 nm (predominantly eosin), 599 nm (hematoxylin), 769 nm (predominantly Cy7), and 880 nm (ir870). The color H&E image (Fig. 7A) was prepared from the unmixed 510 nm and 599 nm images, reproducing the view through the oculars. The images recorded using the 769 nm light channel (predominantly C7 absorbance; Fig. 7B) and 880 nm light channel (ir870 absorbance; Fig. 7C) were processed to provide the unmixed p40 and TTF-1 images (Fig. 7E, F respectively). As evidenced in Fig. 7B, the particularly dark staining ir870 cells selected for this example have noticeable crosstalk into the 769 nm light channel (45% of 880 nm channel absorbance) which is eliminated after unmixing (Fig. 7E). Note that the TTF-1 positive cells in this field of view are more densely stained with ir870 than the larger tumor nuclei in Fig. 5G-I, such that ir870 crosstalk into the Cy7 channel was not as evident in Fig. 5 as in Fig. 7. Unmixed ir870 (Fig. 7F) shows little effect of unmixing due to minimal absorbance of other dyes at 880 nm. Figure 7D shows a color composite produced from combining only the unmixed hematoxylin and TTF-1 images, mimicking a conventional single IHC using hematoxylin counterstain. As expected, p40 staining is absent in the ADC tissue.

## Discussion

Clinical benefits of combining H&E and IHC on the same slide include conservation of small specimens, such as biopsies, to maximize the amount of clinical information obtained from the specimen, and the ability to evaluate protein expression within the full H&E context, bringing to bear the trove of information that H&E based staining provides the pathologist. To realize these benefits, we have developed invisible chromogens, which can be multiplexed with conventional stains, and a dual-camera brightfield microscope system that permits the simultaneous viewing and evaluation of each stain. The absorbance spectra of several invisible chromogens plotted in Fig. 1B show clear spectral separation from the individual hematoxylin and eosin absorbance spectra. The invisible chromogens were developed using CDC technology that simplifies selection of chromogen spectral properties^13^ and allows IHC to be performed ahead of the conventional staining. By performing IHC first, conventional stain is not subjected to IHC reagents that might remove the stain (e.g. eosin removal by aqueous solutions or a variety of non-aqueous solvents), and CDCs are stable to subsequent conventional staining conditions due to the robust covalent attachment.

Simultaneous H&E and IHC is shown in Fig. 3, panels A and B, for the simple case of pancreatic FFPE tissue stained for synaptophysin by IHC using the invisible Cy7 CDC. Using the dual-camera microscope system (Fig. 2), the color camera records the broadband visible light illumination of the H&E stain (Fig. 3A) and the monochrome camera records the NIR illumination of the Cy7 CDC (Fig. 3B). Video of the two camera images, presented side-by-side on the computer monitor, allows the pathologist to visually evaluate both staining patterns on the specimen slide while manually translating the slide. In addition, the H&E stain may be directly viewed through the oculars, with the added precaution of placing bandpass filters within the eyepieces to block transmission of invisible light (see Materials and Methods section and Supplementary Methods document). This permits pathologist examination of the H&E and IHC staining patterns in a manner similar to current practice but on a single slide. To aid cell location, the H&E and IHC images can be combined into a single composite image (Fig. 3C), or a video composite image can be viewed while scanning the specimen using an overlay feature of the software (Fig. 3D–G).

Further multiplexing is possible, as the CDCs in Fig. 1B will support at least duplex IHC. Examples are shown in Fig. 4, panels A-C for tonsil FFPE tissue stained with H&E and CD20/CD8 duplex IHC, and panels D-F for colon tumor FFPE tissue stained with H&E and CD3/CD8 duplex IHC. The two IHC stains are viewed alternately on the monochrome camera by switching between two different invisible illumination channels. A supplementary video may be downloaded that shows the adjacent color and monochrome camera images on the computer monitor while manually scanning a tonsil FFPE specimen and switching between 405 and 770 nm LEDs to alternately present the CD20 DCC CDC and CD8 Cy7 CDC staining patterns, respectively (note video resolution is reduced to limit file size). The image overlay feature is also demonstrated.

The colon tumor example demonstrates important advantages of performing H&E and IHC simultaneously on a single slide. It has been demonstrated in colon carcinoma that the distribution of CD3 and CD8 cells in the core tumor and invasive margin is a strong prognostic marker for disease-free and overall survival^19^. Typically, three FFPE sections are required for the analysis—one for H&E, and one each for CD3 and CD8. The location of the invasive margin is identified on the H&E slide and then transferred to the CD3- and CD8-stained slides on which the respective cell densities relative to the margin are measured. However, since the CD3 and CD8 cells are enumerated on sequential (serial) FFPE sections, the tumor periphery is somewhat altered on each slide, and boundaries must be adjusted to account for differences in tumor section orientation and tumor alterations with section depth relative to the H&E slide. While this approximation of the tumor and margin boundaries on the IHC slides has been shown to provide clinically significant results, and application of artificial intelligence (AI) simplifies the process, performing H&E and dual IHC on the same slide removes all uncertainty in transferring the boundary locations.

In addition to H&E stain on FFPE tissue, PAP stain can be combined with IHC on cytological preparations, which may have limited specimen due to low cellularity. Historically, morphology of cervical cells, as revealed by PAP staining, served as the primary method for screening patients for cervical cancer and dysplasia potentially leading to cervical cancer. More recently, cervical cells overexpressing both p16 and Ki-67, identified by IHC, have been shown to add additional benefit in identifying abnormal cells^20^. Combining PAP and Ki-67/p16 duplex IHC, as shown in Fig. 5, panels A-C, enables the evaluation of PAP staining pattern, p16 expression, and Ki-67 expression in every cell, and may provide greater confidence in the final diagnosis.

Mucicarmine special stain is often used to help identify ADC in NSCLC FFPE tissue by the pink coloration due to increased mucin production relative to SCC. TTF-1 and p40 IHC are also employed to distinguish ADC and SCC and duplex IHC for these two markers combined with mucicarmine special stain should re-enforce the carcinoma assignment on a single slide. Agreement between duplex IHC and mucicarmine special stain is evident in Fig. 5, panels D-F (SCC) and panels G-I (ADC) thereby providing greater confidence in their designations of SCC and ADC. Pink mucin staining is minimal in the SCC specimen (Fig. 5D) which also shows p40 positive cells (Fig. 5E), and mucin production is evident in the ADC specimen (Fig. 5G) in agreement with the presence of TTF-1 positive tumor cells (Fig. 5I).

The dual-camera imaging system is compatible with a pathologist’s manual evaluation of specimens at the microscope since the H&E color camera and monochrome CDC biomarker videos can be viewed simultaneously on a computer monitor while scanning the specimen. The highly corrected 20X microscope objective and LED illuminators used on the dual-camera system can provide both camera images in focus at video rates above 30 fps for the visible and LED illumination between 385 and 770 nm, which includes the HCC, DCC, and Cy7 CDCs. With filtered tungsten illumination, simultaneous focus and similarly high video rates are achieved for the visible, 769 nm (Cy7), and 880 nm (ir870) illumination channels (refer to Supplementary Methods document for detail on chromatic aberration correction and exposure times). Multispectral imaging, using only the monochrome camera, can replace the dual-camera system, if interactive manual scanning of whole slide specimens is not needed, or for further documentation, archiving, and quantification of selected microscope fields. In multispectral imaging, narrowband visible light channels are utilized in addition to the invisible light channels to image both the visible conventional stains and CDCs. Image processing can be applied to multispectral images to remove spectral crosstalk and provide images of individual chromogens and stains with reduced or eliminated interference from other chromogens and stains, as shown in Figs. 6 and 7. Even though the unprocessed images of the colon CD3 stain in Figs. 4E and 6D can be visually evaluated, quantification of CD3 staining can be improved by removing the faint nuclear stain, visible in Fig. 6D, to produce the unmixed image in Fig. 6G that is more effectively segmented for cell counting, measuring spatial relationships, and quantifying expression levels. Even the eosin image (Fig. 6A) is improved by removing faint hematoxylin crosstalk as seen in Fig. 6I. While spectral crosstalk between the Cy7 and HCC CDCs is not visually apparent in the processed images, increasing the number of multiplexed IHC targets or confining the CDCs to a smaller spectral range can increase spectral overlaps between dyes. This was seen in the mucicarmine special stain example (Fig. 7, panels A–F) in which the special stain absorbance in the blue and UV wavelengths (Fig. 1A) required duplex IHC to use two NIR absorbing CDCs, resulting at times in noticeable bleeding of the ir870 CDC absorbance into the Cy7 channel. Image processing to unmix the TTF-1 and p40 multispectral images shows complete removal of the TTF-1 staining in the p40 image (Fig. 7, panel E vs panel B) to reveal the expected lack of p40 staining (Fig. 7E) in this ADC NSCLC specimen.

In multispectral imaging, color images of the conventional stains are generated by combining images recorded at two or more visible illumination bands positioned near the peak absorbance of individual dyes comprising the conventional stain. Figures 6C and 7A show H&E color composite images created from images recorded under illumination near the eosin and hematoxylin absorbance peaks for the colon and ADC NSCLC specimens, respectively, and provide good reproductions of images recorded with the color camera. When the eosin, hematoxylin, and chromogen images are unmixed, composite images can be prepared from any combination of these component images, such as in Fig. 7D in which only the hematoxylin and TTF-1 images are combined to provide a representation equivalent to a single TTF-1 IHC with hematoxylin counterstain. Color composite images can also serve to highlight aspects of the IHC staining, such as co-expression, as shown in Fig. 6F in which CD3 and CD8 have been pseudo-colored magenta and cyan, respectively, producing blue in regions of CD3/CD8 co-expression. Note that cyan cells (CD8 only) are not apparent in the figure since any T-cells expressing CD8 should also co-express CD3, thereby producing the blue coloration.

Not all conventional stains are suitable for multiplexing with invisible IHC. These include black and some brown stains that absorb across the UV, visible, and NIR spectrum, such as iron-hematoxylin complex staining of elastin in Verhoeff’s Van Gieson stain. Invisible IHC can still be used with these stains, but the black stains and pigments will be visible in the monochrome UV and NIR images as well as the visible images. Yellow stains may also obscure the UV and blue portions of the spectrum, as seen here for the tartrazine component of the mucicarmine special stain, limiting invisible chromogens to the NIR.

From the above examples, other applications of invisible chromogens may be imagined. One of these applications would be to augment AI evaluation of H&E stained tissues to increase diagnostic sensitivity and specificity. While AI can come close to, or in some cases match or exceed pathologist interpretation^21^, AI might be improved by enhancing certain cellular, stromal, and overall tissue features with invisible IHC, or identifying tumor-specific markers, providing additional information for diagnosis and correlation with outcomes.

In summary, conventional histological and cytological stains can be combined with IHC on the same slide through the use of invisible chromogens. Clinical benefits include conserving precious specimen material and providing IHC information within the full morphological context and with additional cell specific information that H&E, PAP, and other conventional stains provide. Conventional visible stains and invisible IHC stains can be viewed and evaluated in real-time using a dual camera approach, or multispectral imaging and image processing can be utilized for spectral unmixing, quantitative analysis, and composite image formation.

## Supplementary information

Supplementary Methods

Video demonstration of dual-camera specimen evaluation

## Data Availability

Data generated as part of this study but not presented in the manuscript or supplementary material is available from the corresponding author upon reasonable request.
