# Supplementary material for: Conventional histological and cytological staining with simultaneous immunohistochemistry enabled by invisible chromogens
Source: Lab Invest. 2021 Dec 28;102(5):545–53. doi: 10.1038/s41374-021-00714-2 (PMC9042701; doi:10.1038/s41374-021-00714-2)
Supplement: Supplementary file 1 — Supplementary Methods [file 41374_2021_714_MOESM1_ESM.docx]

**Supplementary Methods**

***Chromogens.*** The chloro-Cy7 chromogen, Ir870, was synthesized as follows.

**2-chlorocyclopentane-1,3-dicarbaldehyde.** To a 100 ml round bottom flask was added 16 ml anhydrous DMF and 16 ml dichloromethane (DCM) and the flask placed on an ice bath with stirring.  To the flask was then added 14.5 ml POCl_3_ (3.8 eq., 155 mmol) in 14 ml DCM and the reaction stirred for 1 hr.  The reaction was allowed to come to room temperature and 3.43 g of cyclopentanone (1.0 eq., 40.8 mmol) in 10 ml of DMF was added over 5 minutes.  The reaction was placed on an oil bath at 65 C^o^ for 3 hr.  After cooling, the reaction was poured onto 80 g of ice and made basic with the careful addition of 10 M NaOH.  The dialdehyde precipitate was collected by filtration and washed 3x with cold water.  Drying under vacuum afforded 5.1 g (77% yield) of the pure dialdehyde.

**6-(1,1,2-trimethyl-1H-3l4-benzo[e]indol-3-yl)hexanoic acid**. To a 100 ml round bottom pressure vessel was added 35 ml dichlorobenzene, 5.0 g 1,1,2-trimethyl-1H-benzo[e]indole (1.0 eq., 23.9 mmol) and 7.0 g 6-bromohexanoic acid (1.5 eq., 35.8 mmol). The sealed flask was placed on an oil bath at 110 °C for 16 hours. After cooling to room temperature, the product was collected by filtration then washed 2x with dichlorobenzene and 2x with ether. The product was dried under vacuum to give 3.56 g (46% yield) of the acid as gray crystals.

**N-(2-(2-(2-(2-azidoethoxy)ethoxy)ethoxy)ethyl)-6-(1,1,2-trimethyl-1H-3l4-benzo[e]indol-3-yl)hexanamide.** To a solution of 1.0 g 6-(1,1,2-trimethyl-1H-3l4-benzo[e]indol-3-yl)hexanoic acid (1.0 eq., 3.22 mmol) in 20 ml of anhydrous DMF was added 990 mg of N,N′-disuccinimidyl carbonate (DSC) (1.2 eq., 3.67 mmol) and 560 mg of 4-dimethylaminopyridine (DMAP) (1.5 eq., 4.83 mmol) the reaction blanketed with nitrogen and stirred at room temperature until ester formation was complete as determined by HPLC (20 minutes). 915 g of 2-(2-(2-(2-azidoethoxy)ethoxy)ethoxy)ethan-1-amine (1.3 eq, 4.19 mmol) was then added and the reaction stirred at room temperature under nitrogen for 18 hours. Preparative HPLC followed by lyophilization afforded 1.13 g (67 % yield) of the pure azide.

**6-(2-((E)-2-((E)-3-((E)-2-(3-(1-azido-13-oxo-3,6,9-trioxa-12-azaoctadecan-18-yl)-1,1-dimethyl-1,3-dihydro-2H-benzo[e]indol-2-ylidene)ethylidene)-2-chlorocyclopent-1-en-1-yl)vinyl)-1,1-dimethyl-1H-3l4-benzo[e]indol-3-yl)-N-(2-(2-(2-(2-azidoethoxy)ethoxy)ethoxy)ethyl)hexanamide.** In a 20 ml amber vial was taken 80 mg N-(2-(2-(2-(2-azidoethoxy)ethoxy)ethoxy)ethyl)-6-(1,1,2-trimethyl-1H-3l4-benzo[e]indol-3-yl)hexanamide (2.0 eq., 0.15 mmol), 12 mg 2-chlorocyclopentane-1,3-dicarbaldehyde (1.0 eq., 0.076 mmol) and 19 mg of anhydrous sodium acetate (3.0 eq., 0.23 mmol) in 7 ml of absolute ethanol. The vial was purged with nitrogen, sealed and placed on an oil bath at 70 °C for 90 minutes. Preparative HPLC followed by lyophilization afforded 44 mg (49 % yield) of the pure cyanine heptamethine dye.

***Immunohistochemistry (IHC) procedures.***  Semi-automated multiplexed detection was performed on a Ventana (VMSI) DISCOVERY Ultra system using the DISCOVERY Universal Procedure to create protocols for the single biomarker IHC and multiplex IHC. In general, IHC was performed at 37°C, unless otherwise noted, and Reaction Buffer wash solutions were diluted from 10x concentrate (cat. no. 950-300). A slide-mounted paraffin section was de-paraffinized by warming the slide to 70°C for 3 cycles, each 8 min long. Antigen retrieval was performed by applying Cell Conditioning 1 (VMSI Cat. no. 950-124) and warming the slide to 94°C for 64 min. Staining of each biomarker was performed sequentially. Steps in each staining reaction included adding 0.1 ml primary antibody targeting that biomarker and incubating for 16-32 min, washing in Reaction Buffer to remove unbound antibody, adding 0.1ml anti-species antibody targeting the primary antibody (either anti-mouse or anti-rabbit) conjugated to peroxidase and incubating for 8 min, washing with Reaction Buffer, manually adding 0.1 ml tyramide-modified dibenzocyclooctyne (tyramide-DBCO) and incubating for 4 min, adding 0.1 ml of 0.01% H2O2 in borate buffer, pH 8.5 and incubating for 32 min. DBCO deposition steps were followed by washing in Reaction Buffer, manually adding 0.1 ml azide-modified chromogen and incubating for 32 min, and washing in Reaction Buffer. If multiplex IHC, the slide was incubated with Cell Conditioning 2 (VMSI Cat# 950-123) at 100°C for 8 min, followed by washing in Reaction Buffer, and continuing with the next biomarker staining steps. At the conclusion of the staining run, the slides were washed with Reaction Buffer and/or a dilute detergent solution (0.2 g Dawn dish liquid (Proctor & Gamble, Cincinnati, OH) in 250 ml water) and rinsed with water. Finally, slides were manually stained with H&E or dehydrated through ethanol and xylene (2 x 80% ethanol, 1 min each; 2 x 90% ethanol, 1 min each; 3 x 100% ethanol, 1 min each; 3 x xylene, 1 min each), at ambient temperature. Primary antibodies and enzyme-antibody conjugates were used at the concentrations, volumes, and incubation times recommended by the manufacturer. Azide-modified CDC reagents and tyramide-DBCO, were added to slides in 100 µL volumes at concentrations ranging between 25 and 1,200 µM in VMSI Discovery TSA diluent (cat no. 000060900). Azide-modified chromogens were applied typically at the same concentration as used for the tyramide-DBCO. The concentrations of CDC solutions reflected their peak absorbance extinction coefficients, and biomarker expression levels, and were typically 400 µM for HCC, 600-800 µM for DCC, 50-300 µM for Cy7, and 50-200 µM for ir870 CDCs.

***Immunocytochemistry (ICC) procedure*.** ICC on cervical specimen pools was performed according to the Ventana CINtec *PLUS* Cytology protocol (package insert) except as noted. CINtec *PLUS* Cytology detection was performed on a Ventana BenchMark Ultra system using the CINtec *PLUS* Cytology cocktailed (p16/ki-67) primary antibodies and detection reagents. In general, ICC was performed at 36°C, unless otherwise noted, and Reaction Buffer wash solutions were diluted from 10x concentrate. Antigen retrieval for cervical specimen pools was performed by applying Cell Conditioning 1 and warming the slide to 75°C for 4 min, then increasing the temperature to 100°C for 24 minutes. Since the CINtec PLUS Cytology antibodies are cocktailed together, incubation with both primary antibodies was performed concurrently, followed by washing in Reaction Buffer to remove unbound antibody. Incubation with anti-species antibodies was performed sequentially targeting the primary antibody (either anti-mouse or anti-rabbit) conjugated to peroxidase. Note that peroxidase-antibody conjugate was used in both staining steps unlike the CINtec procedure which used a peroxidase-antibody conjugate for DAB and an alkaline phosphatase-antibody conjugate for Fast Red. DCC and Cy7 CDCs were substituted for the conventional DAB and Fast Red chromogens, using tyramide DBCO and azide-modified DCC and Cy7. The chromogen deposition portion of the protocol followed the procedure described above for multiplex IHC with CDCs except that the Cell Conditioning 2 incubation at 100°C for 8 min was replaced with addition of 0.2 ml of 30% hydrogen peroxide and incubation at 36ᴼ C for 32 min. The hydrogen peroxide step was performed twice to ensure removal of any peroxidase activity remaining after the first chromogen deposition. At the conclusion of the staining run, the slides were washed with a dilute detergent solution (0.2 g Dawn dish liquid in 250 ml water) and rinsed with water. Conventional PAP staining was performed immediately while slides were still wet.

***Conventional histological and cytological staining procedures.***  H&E staining was performed manually after IHC as follows. If the IHC specimen went through a final dehydration in xylene, then the specimen slide was re-hydrated by soaking in 100% ethanol for 1 min, 90% ethanol for 1 min, 80% ethanol for 1 min, and water for 1 min. Slides were then soaked in Ventana HE 600 Hematoxylin solution (order code 07024282001) or Richard-Allan Hematoxylin I (ThermoFisher, Kalamazoo, MI) for 2 min, water for 2min, Ventana H&E 600 Differentiating solution (order code 06544339001) for 1 min, water for 1 min, Ventana H&E 600 Bluing solution (order code 06544347001) for 1 min, water for 1 min, 95% ethanol for 30 s, Ventana HE 600 Eosin solution (order code 06544304001) for 1 min, 70% ethanol for 1 min, twice in 100% ethanol for 1 min each, and 3 times in xylene for 1 min each. Slides were then allowed to dry briefly and mounted with Richard Allan Scientific Cytoseal XYL (ThermoFisher Scientific), covering with a type 1.5 coverslip, or mounted on a Sakura Finetek USA (Torrance, CA) Tissue-Tek Film Automated Coverslipper.

PAP staining was performed manually after ICC as follows with agitation at each step. The damp slides were soaked in distilled water for 1 minute, Richard-Allan Hematoxylin I for 30 seconds, distilled water twice for 15 seconds each, Richard-Allan Clarifier 1 (ThermoFisher Scientific) for 30 seconds, distilled water for 30 seconds, Richard-Allan Bluing Reagent (ThermoFisher Scientific) for 30 seconds, 50% ethanol for 30 seconds, 95% ethanol for 30 seconds, Richard-Allan Scientific Cyto-Stain (ThermoFisher Scientific) for 1 minute, twice in 95% ethanol for 30 seconds each, three passes through three clean baths of 100% ethanol for 30 seconds each, three passes through xylene, two for 1 minute each and the last for 3 minutes. The PAP-stained slides were then coverslipped as described for H&E staining.

Mucicarmine Special Staining was performed manually after IHC using a commercial reagent kit (cat no. ab150677; Abcam, Cambridge, MA) following manufacturer instruction. This included soaking slides 3 min in hematoxylin solution, washing in water, soaking slides 30 s in bluing reagent, washing in water, soaking slides in mucicarmine solution for 10 min, washing slides in water, soaking slides in tartrazine solution for 1 min, rinsing slides in several changes of ethanol, and soaking slides in xylene. Slides were then drained and mounted as described for H&E staining.

***Dual-camera microscope system.*** Figure 2 shows a schematic drawing of the dual-camera microscope system that permits simultaneous viewing of visible conventional stains and invisible IHC chromogens. Olympus BX-51 and BX-63 microscopes (Olympus, Waltham, MA) were used with UPLXAPO 20X (NA 0.80) and UPLXAPO 10X (NA 0.4) objectives. Referring to the figure, visible illumination is provided by an Olympus 100 W tungsten halogen lamp (A; Olympus U-LH100) with a hot mirror (B) transmitting light between 420 nm and 690 nm (Newport Corp., Irvine, CA; cat. no. 10HMR-0), mounted in a Sutter Lambda 10-3 10-position filter wheel (C; Sutter Instruments, Novato, CA). A color-balancing filter (FGT165 filter, Thorlabs, Newton NJ USA) may also be mounted after the hot mirror to enhance the blue end of the spectrum to improve direct viewing and color camera white balance. Far-blue/UV and far-red/near-IR light is provided by a second illumination source (D) comprising a pE-4000 16-channel LED illuminator (CoolLED, Andover, UK) and Lumencor Spectra X LED light engines (Lumencor, Inc., Beaverton, OR), or an additional Olympus 100 W tungsten halogen lamp, integral IR-blocking filter removed, combined with a filter wheel containing a collection of single bandpass filter sets. To further reduce illumination bandwidth, each Lumencor LED was filtered with a single bandpass optical filter. LED and filter characteristics are provided in Supplementary Table 1, together with monochrome camera exposure times and resulting video frame rates for each invisible illumination channel. Visible illumination (A) and invisible illumination (D) are combined in a pE Combiner (CoolLED) containing a dichroic mirror (E1) with custom coating transmitting light between 420 and 700 nm and reflecting light below 420 nm and above 700 nm (Chroma Technology Corp., Bellows Falls, VT USA ) oriented 45ᴼ to each illumination source. When using LEDs, the invisible illumination source (D) was expanded by combining multiple LED sources using 3mm liquid light guides and one or more light guide combiners (Lumencor, Inc.) ahead of the pE-Combiner. After entering the brightfield illumination port of the microscope, the combined visible and invisible light passes through the specimen slide and microscope objective to the camera port and the dual-camera mount (2SCM1-DC; Thorlabs). A dichroic mirror (E2) within the dual-camera mount, with the same reflective coating as the combiner dichroic mirror splits invisible from visible light, transmitting the visible light to the color camera (G; Kiralux CS505CU, Thorlabs), via a visible light transmitting filter (F; integral to camera) and a 435 nm long pass filter (Newport, 10CGA-435). Invisible light is reflected at the beamsplitter to the monochrome camera (I; Kiralux CS505MU, Thorlabs), via a filter (H) transmitting light below 420 nm and above 700 nm (custom ET560/280 notch filter, Chroma Technology Corp). Both cameras use the same underlying 2448 x 2048 pixel CMOS sensor allowing precise alignment of the two cameras using the translational and rotational adjustments (J) within the 2-camera mount. Thorcam software (Thorlabs) provided control of live video from each camera, image overlays, and single frame image acquisition. In addition to the poor transmission of near UV light through the microscope optics, eye protection from invisible light was provided by custom barrier filters (K; ET560/280m, Chroma Technology Corp) inserted in the reticle space within the microscope eyepieces. The dichroic beamsplitter (E1) and eyepiece barrier filters (J) are designed such that broadband visible light from the standard brightfield microscopy light source (A; Olympus 100 W tungsten halogen lamp) can reach the eye while light from the invisible light channels (D) cannot. This provides the operator with the same viewing experience afforded by a standard brightfield microscope while providing protection from invisible illumination. If ocular viewing is not required and viewing color images of the conventional stains on the computer monitor is sufficient, the oculars can be replaced with a tube lens (Olympus part U-TLU) to eliminate the direct viewing option and the need for the eyepiece barrier filters.

Simultaneous viewing of both visible stains and invisible chromogens on the dual-camera microscope system, from the UV to the NIR, benefits from microscope optics with a high level of chromatic aberration correction. While minor focus adjustments are tolerable, and common for the microscopist while manually scanning slides to compensate for changes in tissue thickness and slide angle, the highly corrected 20X microscope objective ( Olympus UPLXAPO) provided simultaneous color and monochrome images in good focus with minimal or no Z-axis adjustment (see Supplementary Video). This is particularly important for the overlay images. Focal planes were within 1 µm of each other for illumination between the 385 nm and 770 nm LEDs, and between the 405 nm and 880 nm filtered tungsten light. The 10X UPLXAPO objective proved not as well corrected as the 20X objective, with focus adjustments near 15 µm required between UV and NIR light channels. While manual multispectral imaging, as used here, permits re-focusing between light channel images, the chromatic aberration correction of the 20X objective is particularly advantageous to rapid pulsed-LED illumination (see reference 18 in main article) which suffers from a need to re-focus between images.

Illumination intensity is also an issue in the UV and NIR, due to a reduction in transmission through the microscope optics, light source intensity, and detector sensitivity. Light throughput in a microscope with conventional glass optics decreases below 400 nm to very low levels near 350 nm. Digital camera sensitivity declines in the UV and tapers off into the NIR, and tungsten lamp intensity drops greatly in the UV. Despite this we achieved exposure times near 1 ms or less with LEDs between 385 nm and 770 mm, supporting video rates of 35 frames per second (fps). The lower tungsten lamp intensity still permitted 10 ms or less exposure time in the NIR (769 – 880 nm; 35 fps), but dropping to 140 ms (7 fps) at 405 nm and 1 s (1 fps) at 376 nm. Exposure times and resulting camera frame rates are listed in the table in the Supplementary Methods document. While the 385 nm LED provides good images of the HCC CDC, taking advantage of the chromogen’s 365 nm peak absorbance would require a brighter LED to compensate for low light throughput and detector sensitivity at that wavelength.

| **Supplementary Table 1. Illumination channels for multispectral imaging (MS) and dual-camera real-time imaging (DC)** | | | | | | | |
| --- | --- | --- | --- | --- | --- | --- | --- |
| Illumination source | LED nominal λ, nm | bandpass filter, center λ/FWHM, nm | supplier | dye specificity | Imaging mode | Monochrome camera exposure tme, ms* | Monochrome camera frame video rate, fps** |
| 100 W tungsten halogen lamp |  |  | Olympus |  |  |  |  |
|  |  | 10HMR-0 hot mirror, transmits 420-690nm | Newport Corp. | conventional stain | DC |  |  |
|  |  | 376/30 | Semrock | HCC | DC/MS | 990 | 1 |
|  |  | 405/30 | Chroma Technology | HCC, DCC | DC/MS | 140 | 7 |
|  |  | 510/15 | Semrock | eosin | MS |  |  |
|  |  | 599/13 | Chroma Technology | hematoxylin | MS |  |  |
|  |  | 769/49 | Semrock | Cy7 | DC/MS | 1.8 | 35 |
|  |  | 880/40 | Chroma Technology | ir870 | DC/MS | 10 | 35 |
| pE-4000 light engine |  |  | CoolLED |  |  |  |  |
|  | 385 |  |  | HCC |  | 0.8 | 35 |
|  | 405 |  |  | DCC | DC/MS | 0.39 | 35 |
|  | 770 | - |  | Cy7 | DC/MS | 1.1 | 35 |
| Spectra X light engine |  |  | Lumencor |  |  |  |  |
|  | 390 | 390/22 | Semrock | HCC | DC/MS |  |  |
|  | 513 | 513/22 | Semrock | eosin | MS |  |  |
|  | 620 | 620/19 | Semrock | hematoxylin | MS |  |  |
| *100% light directed to camera port, 20X objective, 50% power for each LED, 8 of 12 power setting for 100W tungsten lamp, brightfield background intensity approxiimately 90% of full scale **Video frame rate (fps) at indicated exposure time | | | | | | |  |
